# All‐in‐one sphincterotome with high rotation performance and freely bendable blade for endoscopic sphincterotomy in patients with surgically altered anatomy (a case series with video)

**DOI:** 10.1002/deo2.70019

**Published:** 2024-10-08

**Authors:** Yasuhito Kunogi, Atsushi Irisawa, Akira Yamamiya, Manabu Ishikawa, Tomoya Sakamoto, Yasunori Inaba, Ken Kashima, Fumi Sakuma, Koh Fukushi, Takumi Maki, Kazunori Nagashima, Yoko Abe, Shuichi Kitada, Akane Yamabe, Keiichi Tominaga

**Affiliations:** ^1^ Department of Gastroenterology Dokkyo Medical University School of Medicine Tochigi Japan; ^2^ Department of Gastroenterology Takeda General Hospital Aizuwakamatsu Fukushima Japan

**Keywords:** endoscopic sphincterotomy, ERCP, rotation, sphincterotome, surgically altered anatomy

## Abstract

A new type of sphincterotome released to the market recently has high rotation performance and a freely bendable blade. It is devised to be singly capable of accommodating not only normal anatomy but also cases with surgically altered anatomy. This study was undertaken for clinical evaluation of the usefulness of this new sphincterotome. Eight cases in a reconstructed intestine for which cannulation or endoscopic sphincterotomy (EST) had been performed were extracted from 32 cases for which endoscopic retrograde cholangiopancreatography‐related procedures were performed using the sphincterotome developed during November 2023 through February 2024. The cases were investigated retrospectively. Among these, EST was applied to six cases. Cannulation was performed using the developed sphincterotome in the native papilla in four cases. The primary endpoints were the success rate of cannulation in surgically altered anatomy and the success rate of EST. Secondary endpoints were complications and usability for operators. Usability for operators was evaluated by questionnaire for several items on a 5‐point scale. EST was conducted successfully in all six cases subjected to EST. Mild hemorrhage was observed in one case (17%) as an adverse event after EST. Deep cannulation to the native papilla with the developed sphincterotome was conducted successfully in three cases (75.0%). Evaluation results by operators were 4.4 ± 0.55 for rotation performance, 4.00 ± 0.63 for incision performance, 4.29 ± 0.49 for deep cannulation performance, and 4.07 ± 0.19 for overall evaluation. In conclusion, this developed sphincterotome might be very useful for EST and cannulation in cases with surgically altered anatomy.

## INTRODUCTION

Endoscopic sphincterotomy (EST), a procedure in which a sphincterotome is inserted via the papilla to incise the bile duct of the papillary region from the common channel using a high‐frequency incision tool, has been the basic treatment for endoscopic retrograde cholangiopancreatography (ERCP) since it was reported in 1974.^1^ In general, incision for EST is conducted with a blade placed in the running direction of the bile duct. However, that placement might be difficult in some cases. In such cases, the incision direction is adjusted by bending the blade. Alternatively, the blade direction is adjusted for incision by adding torque on the scope axis. Most cases can be addressed by these methods, but those methods are often difficult for beginners. Some commercially available sphincterotomes have blade direction that can be adjusted by axial rotation of the catheter (capable of rotation). A few reports^2^ have described their usefulness. Nevertheless, their rotation performance is not satisfactory. Moreover, the performance depends greatly upon the operator's skill in not a few cases. Recently, however, there has been a growing number of occasions where balloon‐assisted ERCP is conducted in cases with surgically altered anatomy after gastric cancer, and so forth,^3^ which are often difficult to handle with a normal sphincterotome. The positional relation between the bile duct and the pancreatic duct is reversed, unlike normal anatomy in the endoscopic approach in surgically altered anatomy by other than Billroth I. Therefore, reverse sphincterotomes for such cases are commercially available. Nevertheless, they lack rotation capacity and their flexibility for incision is not high. Because direct‐view balloon endoscopes are used in such cases, deep cannulation with a normal ERCP catheter is often difficult.^4^ One can imagine that a catheter with rotation capacity is useful for deep cannulation in the bile duct.

A sphincterotome has been developed and released to the market recently. The salient characteristic of this device is that the rotation performance of the blade part is extremely high, enabling incision at both knife positions of Push and Pull, resulting from higher tip flexibility. This sphincterotome might ensure cannulation in the bile duct in surgically altered anatomy and might enable effective and safe papillotomy. This investigation was conducted to evaluate the clinical feasibility and safety of this newly developed sphincterotome in cases with surgically altered anatomy, furthermore, this is the first study to report ERCP‐related procedures using this newly developed sphincterotome.

## PROCEDURE OR TECHNIQUE

### Study design

The Medical Ethics Committee of our institution (R‐80‐1J) approved this study, which was conducted at Dokkyo Medical University and was registered with the University Hospital. Medical Information Network (UMIN) Clinical Trials Registry (UMIN000054959). A means to opt out was provided instead of informed consent: research subjects were notified and granted the opportunity via our website to refuse publication of their research information. The primary endpoints were the success rate of cannulation in surgically altered anatomy and the success rate of EST. The secondary endpoints were complications and usability for operators.

### Patients

Eight cases with surgically altered anatomy in which cannulation or EST had been performed were extracted from 32 cases in which ERCP‐related procedures were performed from November 2023 through February 2024 using the sphincterotome developed recently at our hospital and Takeda General Hospital. The cases were investigated retrospectively. Among these, EST was applied to six cases, including one case of endoscopic pancreatic sphincterotomy. Cannulation was performed with the developed sphincterotome in the native papilla in four cases. Native papillae were defined as cases in which EST or biliary stent placement had not been performed. The exclusion criteria were set as those cases in which the patients without surgically altered anatomy the subjects withdrew research participation after information disclosure and those cases that were judged as inappropriate to be included in this study by researchers: no case was excluded (Figure S1).

### ERCP procedure

The procedures were carried out using double‐balloon enteroscopy (EI‐580BT; Fujifilm), single‐balloon enteroscopy (SIF‐H290S; Olympus Medical Systems Corp.), side‐viewing therapeutic duodenoscopes (JF‐260V; Olympus Medical Systems Corp.), and the developed sphincterotome (ENGETSU; Kaneka Corp.). A standard injection catheter (MTW ERCP‐catheter, filiform; MTW Endoskopie Manufaktur, Wesel, Germany) was used. Also, a GW of angle‐tip (VisiGlide 2; Olympus Medical Systems Corp. and Endoselecotor; Boston Scientific Corp.) was used. The method used for deep bile duct cannulation was wire‐guided cannulation or contrast cannulation, based on the operator's decision. For the cannulation, time is set as the time from the front view at the papilla to when deep cannulation is confirmed on the contrast image. The operation time is set as the time from the front view at the papilla to when the endoscopic operation is completed. The operators were four experts with experience of not less than 500 cases of ERCP. If necessary, EST was performed; a high‐frequency incision tool (VIO300S; Erbe Elektromedizin GmbH) was used.

### Newly developed sphincterotome

**FIGURE 1:**
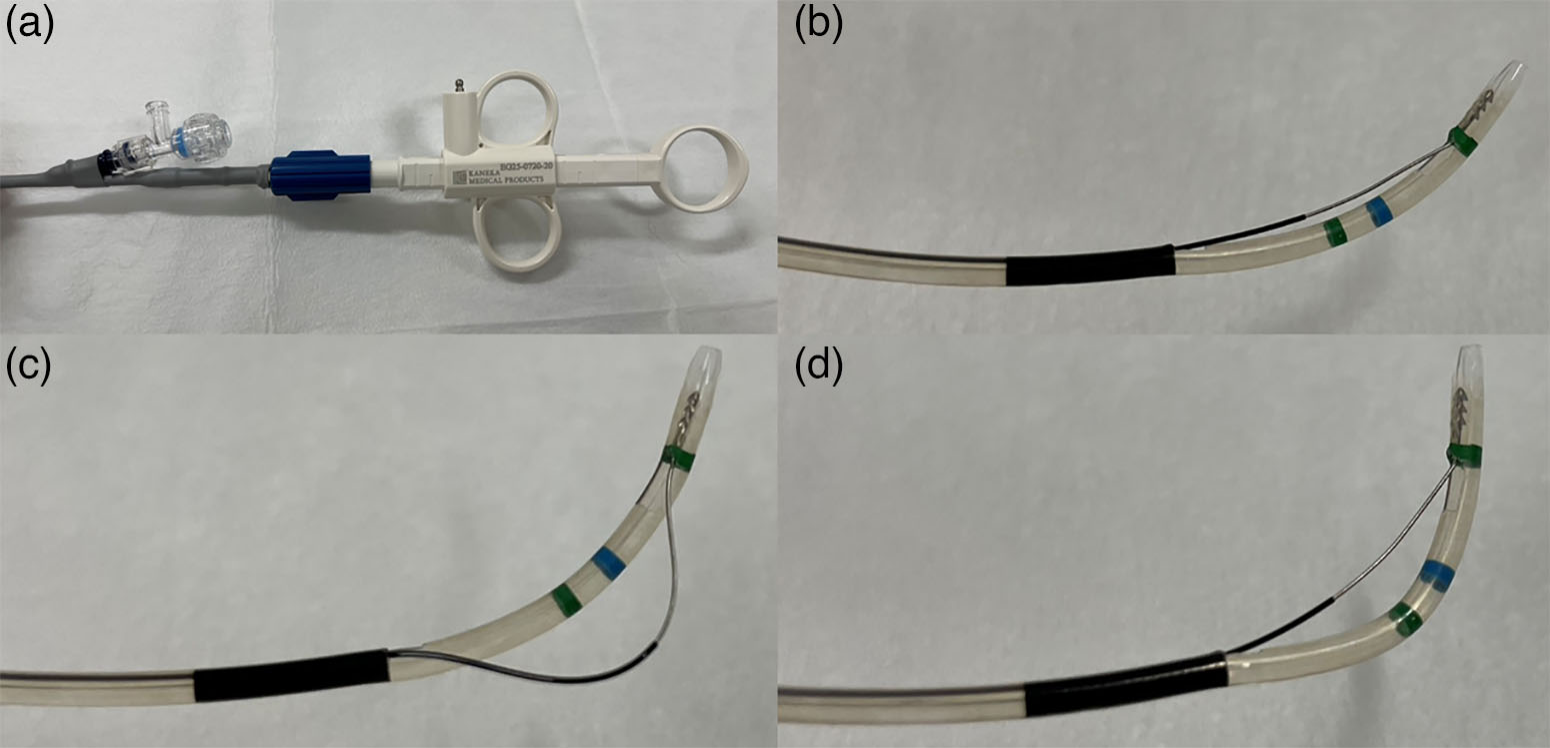
Images of the handle and tip of a newly developed sphincterotome. (a) The handle, (b) the normal shape of the tip, (c) the push shape of the tip, and (d) the pull shape of the tip.

The newly developed sphincterotome has a 7 mm tip length, 25 mm knife length, 7.6 Fr maximum diameter of the insertion part, 2000 mm effective length, and 0.025 inches of compatible guidewire diameter. The rotation performance of the blade part is extremely high, enabling incision at both knife positions of Push and Pull, resulting from the greater tip flexibility. It is also characteristic that the blade is semicircular at the push position and resembles a conventional knife in the pull position (Figure 1 and Video S1).

### Definitions of complications

Definitions of complications were based on those described by Cotton et al. Each major type of complication was graded as mild, moderate, or severe.^5^

#### Bleeding

Hemorrhage was defined as clinical, not just endoscopic evidence of bleeding. Mild hemorrhage was defined as a hemoglobin drop of 3 g/L or less and not requiring transfusion, moderate as requiring a transfusion of four units or fewer without needing angiographic intervention or surgery, and severe as requiring five or more units or intervention.

#### Pancreatitis

Pancreatitis was defined as abdominal pain persisting for at least 24 h associated with serum amylase of greater than three times the upper limit of normal. Mild pancreatitis was that which required admission for three or fewer days, moderate for 4–10 days, and severe for more than 10 days.

#### Perforation

Perforation was defined as air or contrast leak into the peritoneal or retroperitoneal cavity recognized either at the time of ERCP or on subsequent imaging. Mild perforation was defined as a possible or very small leak treated using fluids and nasogastric decompression for 3 or fewer days. Moderate was defined as any definite perforation treated medically for 4–10 days. Severe was defined as medical treatment for more than 10 days or any intervention (surgical or radiological).

#### Cholangitis

Cholangitis was characterized as a septic illness lasting more than 24 h in an obstructed patient without any other clear source of infection. Mild was defined as lasting less than 48 h, moderate lasting 3 or more days of hospital treatment, or endoscopic or percutaneous intervention. Severe cholangitis was defined as septic shock or that requiring emergent surgery.

All other complications were graded based on the length of hospitalization and the need for surgery. Mild was defined as a prolonged stay of three or fewer days, moderate 4–10 days severe greater than 10 days, and/or requiring intensive care or surgery.

Mortality was defined as either ERCP‐specific or death from all causes within 30 days. Death was inferred as ERCP‐specific if the death was secondary to a complication related to the procedure, occurring in organs traversed or treated by ERCP and with symptoms developing within 30 days.

### Definition of usability for operators

Usability for operators was evaluated for 12 items listed below on a five‐point scale by questionnaire: tip shape, performance of insertion into the endoscope, deep cannulation performance, contrast performance, rotation performance, Pull cut, Push cut, knife visibility, incision performance, GW insertion performance, GW operability, and overall evaluation for the sphincterotome. The points were defined as the following: 1, useless; 2, poor; 3, average; 4, good; 5, superior.

### Definitions of outcomes

The success rate of EST is defined as the percentage of EST completion using the newly developed sphincterotome singly.

The success rate of deep bile duct cannulation is set as the percentage of deep bile duct cannulation using the newly developed sphincterotome singly by contrast cannulation or wire‐guided cannulation in bile duct cannulation. The results were recorded as a failure when cannulation was performed replacing other catheters, and so forth, or using methods such as the pancreatic duct GW method.

### Definitions of emergency and elective cases

Emergency cases are defined as cases in which ERCP was performed within 24 h after the diagnosis for which detailed examination or treatment with ERCP was required. Elective cases are defined as other cases.

## RESULTS

### Patients

We examined data from eight patients (four males and four females) with a median age of 69 years, of which three cases were selective and five cases were emergencies. Target diseases were three cases of choledocholithiasis, one of pancreatic cancer, one of hilar cholangiocarcinoma, one of hepatoma, one of lymph node metastasis of gastric cancer, and one of intraductal papillary mucinous neoplasm, with native papilla in four cases. Surgical reconstruction was done by Billroth‐I in two cases, Billroth‐II in three cases, and Roux‐en‐Y in three cases (Table S1). Table 1 presents details of the cases.

**TABLE 1:** List of eight patients.

No.	Sex	Age	Target disease	Indication for ERCP	Emergency procedure	Surgical reconstruction	Scope	Benign/Malignant	Papilla	PAD	First cannulation device	Cannulation success	Cannulation method	Cannulation time	Adverse event
1	Female	45	LN metastasis of gastric cancer	Acute cholangitis	Yes	R‐Y	DBE	Malignant	Post‐ PS placement	No	ENGETSU	Yes	Along PS	3	No
2	Female	82	Hilar cholangiocarcinoma	Obstructive jaundice	No	R‐Y	DBE	Malignant	Post‐ PS placement	No	ENGETSU	Yes	Along PS	4	No
3	Female	68	Hepatoma	Acute cholangitis	Yes	B‐II	DBE	Malignant	Post‐ EST/ PS placement	No	ERCP catheter^＊^	Yes	Along PS	11	No
4	Female	79	IPMN	Detailed examination	No	B‐I	DS	Benign	Native	No	ENGETSU	Yes	WGC	20	No
5	Male	77	Pancreatic cancer	Acute cholangitis	Yes	B‐I	DS	Malignant	Native	No	ENGETSU	Yes	WGC	3	Post‐EST hemorrhage
6	Male	70	Choledocholithiasis	Acute cholangitis	Yes	R‐Y	SBE	Benign	Native	No	ENGETSU	No	CGC	10	No
7	Male	64	Choledocholithiasis	Acute cholangitis	Yes	B‐II	DBE	Benign	Native	No	ENGETSU	Yes	WGC	1	No^¶^
8	Male	64	Choledocholithiasis	Stone removal	No	B‐II	DBE	Benign	Post‐ PS placement	No	ENGETSU	Yes	Along PS	1	No

Abbreviations: B‐I, Billroth‐I; B‐II, Billroth‐II; CGC; contrast guided cannulation; DBE, double balloon enteroscopy; DS, duodenoscope; IPMN, Intraductal papillary mucinous neoplasm; LN, lymph node; N/A, not applicable; PAD, periampullary diverticulum; PS, plastic stenting; R‐Y, Roux‐en‐Y; SBE, single balloon enteroscopy; WGC, wire‐guided cannulation.

* The operator initially determined that bile duct cannulation was possible using the usual ERCP catheter, but cannulation was difficult to, do so the catheter was changed to ENGETSU.

^¶^ In this case, ERCP was performed due to severe cholangitis. The patient's vital signs became unstable during the procedure, so EST was not performed and the procedure was completed with only stent insertion.

#### About EST

EST was applied to six cases with surgically altered anatomy. EST was successful in all the cases. An adverse event after EST was mild hemorrhage in one case (16.6%; Table 2). No complications after EST other than mild hemorrhage were observed.

**TABLE 2:** Characteristics of six patients who performed endoscopic sphincterotomy/endoscopic pancreatic sphincterotomy.

Characteristic	Value
Sex, male/female	3/3
Median age, years	73.5 (45–82)
Emergency procedure	3 (50)
Benign/Malignant	3/3
Target disease	
Choledocholithiasis	2 (33.3)
Pancreatic cancer	1 (16.7)
IPMN	1 (16.7)
Hilar cholangiocarcinoma	1 (16.7)
LN metastasis of gastric cancer	1 (16.7)
Indication for ERCP	
Acute cholangitis	3 (50)
Obstructive jaundice	1 (16.7)
Stone removal	1 (16.7)
Detailed examination	1 (16.7)
Surgical reconstruction	
Billroth‐I gastrectomy	2 (33.3)
Billroth‐II gastrectomy	1 (16.7)
Roux‐en‐Y gastrectomy	3 (50)
Native papilla	3 (50)
EST performed	5 (83.3)
EPST performed	1 (16.7)
Adverse events	1 (16.7)
Post‐EST hemorrhage	1 (16.7)

Values are median (range) or *n* (%).

Abbreviations: ERCP, endoscopic retrograde cholangiopancreatography; EPST, endoscopic pancreatic sphincterotomy; EST, endoscopic sphincterotomy; IPMN, intraductal papillary mucinous neoplasm; LN, lymph node.

#### About cannulation

Cannulation was performed in seven cases with surgically altered anatomy using the newly developed sphincterotome, among which four cases were with the native papilla and surgically altered anatomy, including two cases of Billroth‐I, 1 case of Billroth‐II, and one case of Rou‐en‐Y. Deep cannulation was successful in three cases (75%). The median bile duct cannulation time was 6.5 min (1–20 min). No complications cannulation related to cannulation were observed (Table 3).

**TABLE 3:** Characteristics of four cases of cannulation of native papilla.

Characteristic	Value
Sex, male/female	3/1
Median age, years	73.5 (64–79)
Emergency procedure	3 (75)
Benign/malignant	3/1
Target disease	
Choledocholithiasis	2 (50)
Pancreatic cancer	1 (25)
IPMN	1 (25)
Indication for ERCP	
Acute cholangitis	3 (75)
Detailed examination	1 (25)
Surgical reconstruction	
Billroth‐I gastrectomy	2 (50)
Billroth‐II gastrectomy	1 (25)
Roux‐en‐Y gastrectomy	1 (25)
Cannulation success	3 (75)
Cannulation method, WGC/CGC	3/1
Cannulation time, min	6.5 (1–20)

Values are median (range) or *n* (%).

Abbreviations: CGC; contrast‐guided cannulation; ERCP, endoscopic retrograde cholangiopancreatography; IPMN, intraductal papillary mucinous neoplasm; WGC, wire‐guided cannulation.

#### Usability for operators

Tip shape was 3.57 ± 0.34, the performance of insertion into the endoscope was 3.5 ± 0.65, deep cannulation performance was 4.29 ± 0.49, contrast performance was 3.36 ± 0.48, rotation performance was 4.40 ± 0.55, Pull cut was 3.86 ± 0.85, Push cut was 3.83 ± 0.41, knife visibility was 4.14 ± 0.38, incision performance was 4.00 ± 0.63, GW insertion performance was 3.29 ± 1.11, GW operability was 3.36 ± 0.48, and overall evaluation was 4.07 ± 0.19 (Table 4 and Figure S2).

**TABLE 4:** Evaluation score of sphincterotome by operator, *n*.

Evaluation item, *n*	Score, mean ± SD
Tip shape, 7	3.57 ± 0.34
Performance of insertion into the endoscope, 7	3.5 ± 0.65
Deep cannulation performance, 7	4.29 ± 0.49
Contrast performance,7	3.36 ± 0.48
Rotation performance, 5	4.40 ± 0.55
Pull cut, 4	3.86 ± 0.85
Push cut, 6	3.83 ± 0.41
Knife visibility, 7	4.14 ± 0.38
Incision performance, 6	4.0 ± 0.63
GW insertion performance, 7	3.29 ± 1.11
GW operability, 7	3.36 ± 0.48
overall score, 7	4.07 ± 0.19

Guideline for Evaluation Score:

1: useless.

2: poor.

3: average.

4: good.

5: superior.

## DISCUSSION

ERCP has been popular for the diagnosis and treatment of hepatopancreatic diseases. Success rates as high as 90%–95% have been achieved in cases with normal anatomy.^6^, ^7^ However, it has been supposed to be difficult to conduct ERCP‐related treatment in patients with surgically altered anatomy. Balloon‐assisted endoscopy (BAE) was developed in 2001 for the diagnosis and treatment of small intestinal diseases.^8^ Subsequently, since the ERCP‐related procedure using BAE in a patient with surgically altered anatomy was reported in 2005 by Haruta,^9^ currently ERCP‐related procedures in patients with surgically altered anatomy have been performed using BAE. The success rate is improving by virtue of recent progress in BAE. However, in cases with surgically altered anatomy, sometimes the scope cannot reach the target site because the distance to the site is long and the scope is liable to bend, in addition to the effects of the acute angle and adhesion at the intestinal anastomosis. Moreover, even if the scope is able to reach the target site, the papilla is located in the tangential direction, and BAE is not equipped with a forceps elevator, frequently leading to difficulty in bile duct cannulation. The ESGE guidelines state clearly that cannulation is difficult in cases with surgically altered anatomy.^10^ Several reports have described factors leading to failure in cannulation in BAE‐ERCP. For example, reported failure factors include the following: malignant biliary obstruction, first ERCP attempt and Roux‐en‐Y reconstruction by Tanisaka et al.^11^; younger age and Roux‐en‐Y reconstruction by Izawa et al.^12^; and Roux‐en‐Y reconstruction, first‐time short DBE‐ERCP by Uchida et al.^13^ For Roux‐en‐Y reconstruction and the native papilla, it has been agreed that cannulation in the retroflex position can ensure a good visual field at the papilla.^14^, ^15^

As described above, the success rate of ERCP‐related procedures in surgically altered anatomy is regarded as improved also by devising scope operation. Recently it is also anticipated that the development of new catheters will contribute to the improvement of the success rate. For example, Takenaka et al. reported a method by which an indwelling guide wire is placed in the pancreatic duct, and a catheter is put at the pancreatic duct orifice using Uneven Double Lumen Cannula (Piolax Medical Devices Inc.). Then bile duct cannulation is attempted using a guide wire via the other lumen.^16^ Inoue et al. reported that bile duct cannulation succeeded in all cases of bile duct cannulation failure for cases with surgically altered anatomy by pre‐cutting using a scissor‐type knife.^17^

The newly developed sphincterotome used for this study is a device with higher flexibility at the tip, which provides good rotation performance and incision at both knife positions of Push and Pull. This feature supports advanced approaches to the papilla at various positions and leads to an ideal incision in EST. In cases with surgically altered anatomy, especially in cases of Roux‐en‐Y reconstruction, the papilla is located in the tangential direction. Also, BAE is not equipped with a forceps elevator, resulting in difficulty in bile duct cannulation. Even in B‐I reconstruction, the papilla location shifts because of postoperative adhesion and anatomical deformation, frequently leading to difficulty in cannulation. In addition, a common sphincterotome is made for normal anatomy and is therefore designed so that it can incise in the 11 o'clock direction, which is the direction of the bile duct axis when the papilla is viewed in the front. However, in surgically altered anatomy, the EST incision direction is found after confirming the oral protrusion, but the knife wire is not always faced in the direction of the incision. EST is a difficult procedure for which reported techniques include the use of a dedicated, push‐type sphincterotome, modification of the existing knife, incision using a needle‐shaped knife with a plastic stent inserted in the bile duct, rotation of the scope so that the knife part of the sphincterotome matches the incision direction and other techniques.^18^, ^19^ However, safer and more reliable EST is possible even by a trainee if a sphincterotome were more mobile and flexible, without depending on the skill of the operator. Based on this background, this study investigated the usefulness and safety of the newly developed sphincterotome in ERCP in cases with surgically altered anatomy. In the present investigation, EST in cases with surgically altered anatomy was successful in all cases. Deep cannulation in the native papilla was also successful at a probability as high as three of four cases. The factors are, as described earlier, the good rotation performance of the newly developed sphincterotome, as well as the specification of the tip with higher flexibility, which enables incision at both knife positions of Push and Pull. Mild hemorrhage because of incision was observed in 1 case, although there was no severe case. With EST, a risk exists of not only hemorrhage but also perforation if there is an error in the direction of incision,^20^ requiring surgical treatment depending on the situation. The rate of complication is expected to decrease when using the newly developed sphincterotome with which the knife position can be manipulated easily. Another advantage of the newly developed sphincterotome is that it is a catheter that is compatible with both SBE and DBE.

In addition, the results of the evaluation for the newly developed sphincterotome by operators were 4.40 ± 0.55 for rotation performance, 4.00 ± 0.63 for incision performance, 4.29 ± 0.49 for deep cannulation performance, and 4.07 ± 0.19 for overall evaluation. The evaluation results are higher than those for conventional sphincterotomes for all parameters, suggesting good usefulness of the newly developed sphincterotome.

Because this study is a two‐center retrospective study, the fundamental limitation of this study is its small sample size. Another limitation is that the choice of catheter is left to the operator. However, high operability and safety were demonstrated in cases with surgically altered anatomy where ERCP‐related procedures are more difficult than for normal anatomy. Based on findings from this investigation, it is reasonably expected that the new sphincterotome is also useful in cases with normal anatomy. In conclusion, the newly developed sphincterotome might be very useful for EST and cannulation in cases with surgically altered anatomy.

## CONFLICT OF INTEREST STATEMENT

None.

## ETHICS STATEMENT

The Medical Ethics Committee of our institution (R‐80‐1J) approved this study, which was conducted at Dokkyo Medical University and was registered with the University Hospital. Medical Information Network (UMIN) Clinical Trials Registry (UMIN000054959). A means to opt out was provided instead of informed consent: research subjects were notified and granted the opportunity via our website to refuse publication of their research information.

## Supporting information




**Figure S1** Flow diagram showing patient selection criteria.

**Figure S2** Bar graphs of each rating distribution by each operator for a newly developed sphincterotome.

**Table S1** Baseline characteristics of eight patients.

**Video S1** How to use an All‐in‐one sphincterotome with high rotation performance and a freely bendable blade.

## References

[1] Kawai K , Akasaka Y , Murakami K , Tada M , Kohli Y , Nakajima M . Endoscopic sphincterotomy of the ampulla of Vater. Gastrointest Endosc 1974; 20: 148–151. 4825160 10.1016/s0016-5107(74)73914-1

[2] Okamoto T , Sasaki T , Takeda T *et al*. Rotatable sphincterotome as a rescue device for endoscopic retrograde cholangiopancreatography cannulation: A single‐center experience. Clin Endosc 2023; 57: 96–104. 37157962 10.5946/ce.2022.248 PMC10834294

[3] Spadaccini M , Giacchetto CM , Fiacca M *et al.* Endoscopic biliary drainage in surgically altered anatomy. Diagnostics 2023; 13: 3623. 38132207 10.3390/diagnostics13243623 PMC10742737

[4] Shimatani M , Mitsuyama T , Yamashina T *et al*. Advanced technical tips and recent insights in ERCP using balloon‐assisted endoscopy. DEN Open 2024; 4: e301. 38023665 10.1002/deo2.301 PMC10644950

[5] Cotton PB , Lehman G , Vennes J *et al*. Endoscopic sphincterotomy complications and their management: An attempt at consensus. Gastrointest Endosc 1991; 37: 383–393. 2070995 10.1016/s0016-5107(91)70740-2

[6] Freeman ML , Guda NM . ERCP cannulation: A review of reported techniques. Gastrointest Endosc 2005; 61: 112–125. 15672074 10.1016/s0016-5107(04)02463-0

[7] Suissa A , Yassin K , Lavy A *et al*. Outcome and early complications of ERCP: A prospective single center study. Hepatogastroenterology 2005; 52: 352–355. 15816433

[8] Yamamoto H , Sekine Y , Sato Y *et al*. Total enteroscopy with a nonsurgical steerable double‐balloon method. Gastrointest Endosc 2001; 53: 216–220. 11174299 10.1067/mge.2001.112181

[9] Haruta H , Yamamoto H , Mizuta K *et al*. A case of successful enteroscopic balloon dilation for late anastomotic stricture of choledochojejunostomy after living donor liver transplantation. Liver Transpl 2005; 11: 1608–1610. 16315301 10.1002/lt.20623

[10] Testoni PA , Mariani A , Aabakken L *et al*. Papillary cannulation and sphincterotomy techniques at ERCP: European Society of Gastrointestinal Endoscopy (ESGE) clinical guideline. Endoscopy 2016; 48: 657–683. 27299638 10.1055/s-0042-108641

[11] Tanisaka Y , Ryozawa S , Mizuide M *et al*. Analysis of the factors involved in procedural failure: Endoscopic retrograde cholangiopancreatography using a short‐type single‐balloon enteroscope for patients with surgically altered gastrointestinal anatomy. Dig Endosc 2019; 31: 682–689. 30942924 10.1111/den.13414

[12] Izawa N , Tsuchida K , Tominaga K *et al*. Factors affecting technical difficulty in balloon enteroscopy‐assisted endoscopic retrograde cholangiopancreatography in patients with surgically altered anatomy. J Clin Med 2021; 10: 1100. 33800779 10.3390/jcm10051100 PMC7961549

[13] Uchida D , Tsutsumi K , Kato H *et al*. Potential factors affecting results of short‐type double‐balloon endoscope‐assisted endoscopic retrograde cholangiopancreatography. Dig Dis Sci 2020; 65: 1460–1470. 31562611 10.1007/s10620-019-05857-3

[14] Ishii K , Itoi T , Tonozuka R *et al*. Balloon enteroscopy‐assisted ERCP in patients with Roux‐en‐Y gastrectomy and intact papillae (with videos). Gastrointest Endosc 2016; 83: 377–386. 26234697 10.1016/j.gie.2015.06.020

[15] Tanisaka Y , Ryozawa S , Mizuide M *et al*. Biliary cannulation in patients with Roux‐en‐Y gastrectomy: An analysis of the factors associated with successful cannulation. Intern Med 2020; 59: 1687–1693. 32296000 10.2169/internalmedicine.4245-19 PMC7434537

[16] Takenaka M , Minaga K , Kamata K *et al*. Efficacy of a modified double‐guidewire technique using an uneven double lumen cannula (uneven method) in patients with surgically altered gastrointestinal anatomy (with video). Surg Endosc 2020; 34: 1432–1441. 31667613 10.1007/s00464-019-07228-5

[17] Inoue T , Ibusuki M , Kitano R *et al*. Scissor‐type knife precut in balloon enteroscopy‐assisted ERCP for patients with difficult biliary cannulation and surgically altered anatomy (with video). Gastrointest Endosc 2022; 95: 717–722. 34762919 10.1016/j.gie.2021.10.032

[18] Ito K , Masu K , Kanno Y , Ohira T , Noda Y . Ampullary intervention for bile duct stones in patients with surgically altered anatomy. Dig Endosc 2014; 26: 116–121. 24750160 10.1111/den.12250

[19] Siegel JH , Cohen SA , Kasmin FE , Veerappan A . Stent‐guided sphincterotomy. Gastrointest Endosc 1994; 40: 567–572. 7988820 10.1016/s0016-5107(94)70254-3

[20] Pal P , Ramchandani M . Management of ERCP complications. Best Pract Res Clin Gastroenterol 2024; 69: 101897. 38749576 10.1016/j.bpg.2024.101897

