# Supplementary figures and images for: All‐in‐one sphincterotome with high rotation performance and freely bendable blade for endoscopic sphincterotomy in patients with surgically altered anatomy (a case series with video)
Source: DEN Open. 2024 Oct 8;5(1):e70019. doi: 10.1002/deo2.70019 (PMC11461899; doi:10.1002/deo2.70019)

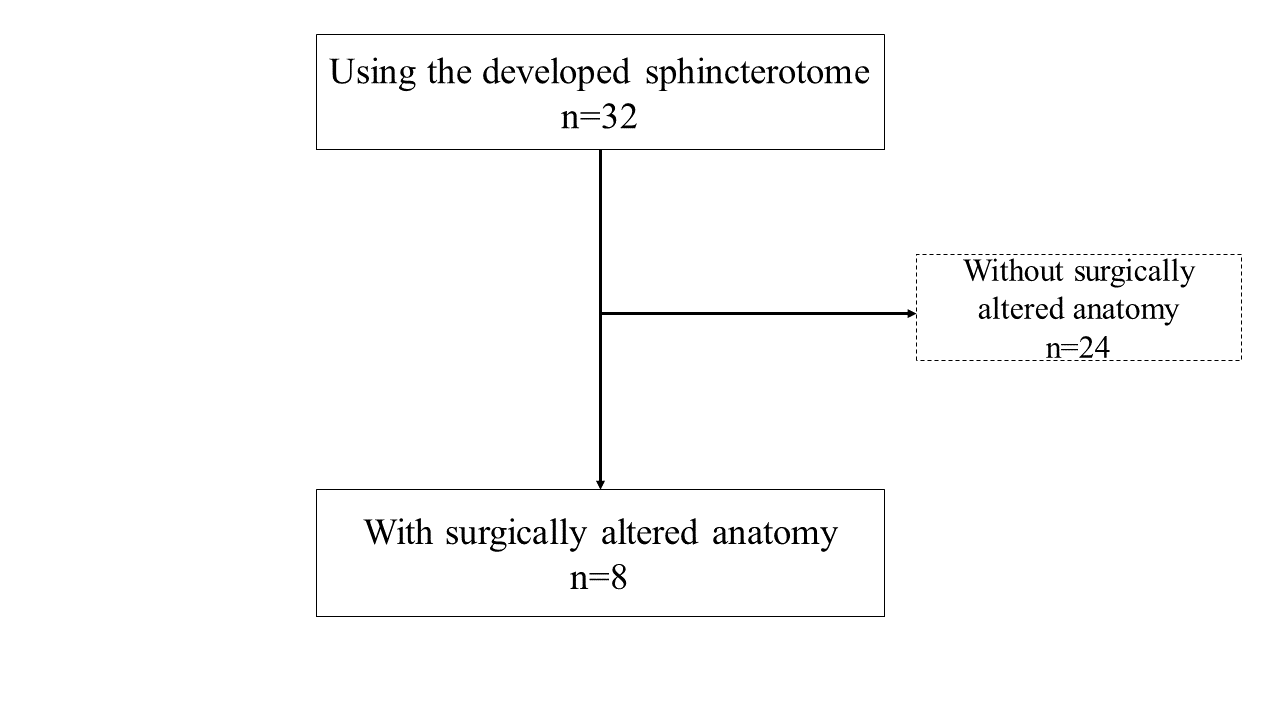

Supplement: Supplementary file 1 — Figure S1 Flow diagram showing patient selection criteria. [file DEO2-5-e70019-s003.png]

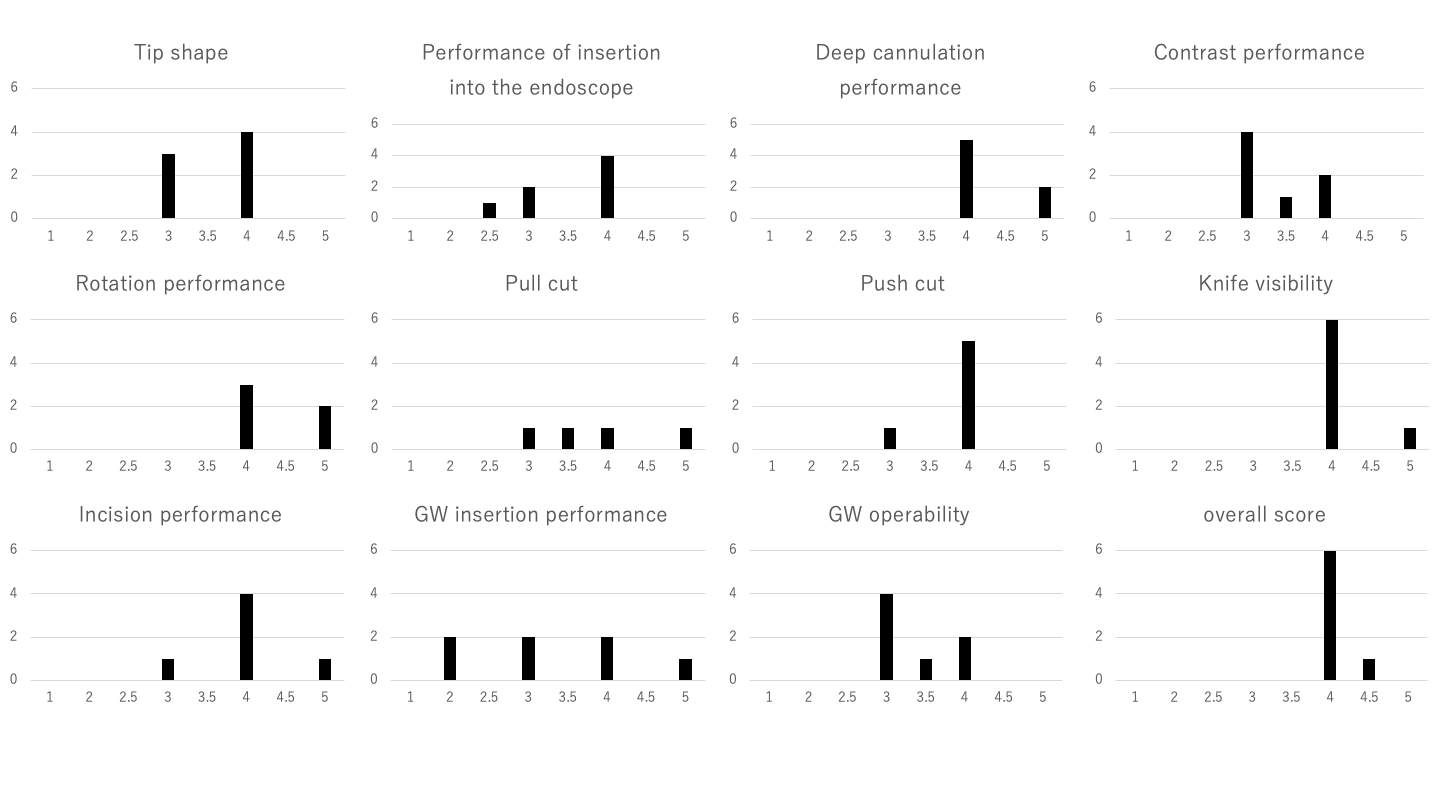

Supplement: Supplementary file 2 — Figure S2 Bar graphs of each rating distribution by each operator for a newly developed sphincterotome. [file DEO2-5-e70019-s004.png]
