# Supplementary material for: All‐in‐one sphincterotome with high rotation performance and freely bendable blade for endoscopic sphincterotomy in patients with surgically altered anatomy (a case series with video)
Source: DEN Open. 2024 Oct 8;5(1):e70019. doi: 10.1002/deo2.70019 (PMC11461899; doi:10.1002/deo2.70019)
Supplement: Supplementary file 3 — Table S1 Baseline characteristics of eight patients. [file DEO2-5-e70019-s001.docx]

　Supplementary Table.1 Baseline characteristics of 8 patients

| Characteristic | value |
| --- | --- |
| Sex, male/female | 4/4 |
| Median age, y | 69(45-82) |
| Emergency procedure | 5(62.5) |
| Benign/Malignant | 4/4 |
| Target diseases |  |
| Choledocholithiasis | 3(37.5) |
| Pancreatic cancer | 1(12.5) |
| IPMN | 1(12.5) |
| Hilar cholangiocarcinoma | 1(12.5) |
| Hepatoma | 1(12.5) |
| LN metastasis of gastric cancer | 1(12.5) |
| Indication for ERCP |  |
| Acute cholangitis | 5(62.5) |
| Obstructive jaundice | 1(12.5) |
| Stone removal | 1(12.5) |
| Detailed examination | 1(12.5) |
| Surgical reconstruction |  |
| Billroth-I gastrectomy | 2(25.0) |
| Billroth-II gastrectomy | 3(37.5) |
| Roux-en-Y gastrectomy | 3(37.5) |
| Papilla |  |
| Native papilla | 4(50) |
| Post PS placement | 3(37.5) |
| Post EST and PS placement | 1(12.5) |
| EST/EPST performed |  |
| EST | 5(62.5) |
| EPST | 1(12.5) |
| Not performed | 2(25.0) |

Values are median(range) or n (%)

Abbreviations: IPMN, Intraductal Papillary Mucinous Neoplasm; LN, Lymph Node; ERCP, Endoscopic Retrograde Cholangiopancreatography; EST, Endoscopic　Sphincterotomy; PS, Plastic stent; EPST, Endoscopic Pancreatic Sphincterotomy.
