# Supplementary material for: All‐in‐one sphincterotome with high rotation performance and freely bendable blade for endoscopic sphincterotomy in patients with surgically altered anatomy (a case series with video)
Source: DEN Open. 2024 Oct 8;5(1):e70019. doi: 10.1002/deo2.70019 (PMC11461899; doi:10.1002/deo2.70019)
Supplement: Supplementary file 4 — Video S1 How to use an All‐in‐one sphincterotome with high rotation performance and a freely bendable blade. [file DEO2-5-e70019-s002.docx]

Supplementary video 1 can be viewed here:

[【DENOP-2024-0144】All-in-one sphincterotome_video1.mp4](https://wiley-my.sharepoint.com/:v:/p/yikegami/ESpi8AyfQ3ZGipKORST0GYkBNRvCiP_Bhoyzr_M9yrog3Q?nav=eyJyZWZlcnJhbEluZm8iOnsicmVmZXJyYWxBcHAiOiJPbmVEcml2ZUZvckJ1c2luZXNzIiwicmVmZXJyYWxBcHBQbGF0Zm9ybSI6IldlYiIsInJlZmVycmFsTW9kZSI6InZpZXciLCJyZWZlcnJhbFZpZXciOiJNeUZpbGVzTGlua0NvcHkifX0&e=c72FCK)
